# Supplementary material for: Mixed-valence insulators with neutral Fermi surfaces
Source: Nat Commun. 2018 May 2;9:1766. doi: 10.1038/s41467-018-04163-2 (PMC5932084; doi:10.1038/s41467-018-04163-2)
Supplement: Supplementary file 1 — Supplementary Information [file 41467_2018_4163_MOESM1_ESM.pdf]

Supplementary Information : "Mixed-valence insulators with neutral Fermi surfaces"

SUPPLEMENTARY NOTE 1

Fermionic composite exciton hopping: Consider the situation where there is a single site with configuration $4f^5 5d^1$, surrounded by sites with configuration $4f^6$. As $U_{\rm ff} \to \infty$, and within slave-boson theory, the single site is identified by $n^{\tilde{f}} = n^b = 1$. At large $U_{df} (\ll U_{ff})$, the lowest energy configuration will be the state where the binding has taken place with the formation of the CE. In order for the fCE to hop, both the holon as well as the $d-$electron have to hop to the neighboring sites via virtual processes. Let us now estimate this hopping amplitude within a controlled approximation. For this purpose, consider the following related Hamiltonian,

$$\tilde{H} = H_d + H_{df} + H_{{\rm t}_f\text{-}{\rm J_H}} + H_{\rm hyb}, \tag{1}$$

where H_d and H_{df} represent the Hamiltonian for the $d-$electrons and the repulsive density-density interactions with strength U_{df} between the d and $f-$electrons respectively. We have introduced $H_{{\rm t}_f\text{-}{\rm J_H}}$ as an effective model for the $\tilde{f}-$hole with,

$$H_{{\rm t}_f\text{-}{\rm J_H}} = P_{\rm G}\Big[- t_f \sum_{\langle \mathbf{r}\mathbf{r}'\rangle} \tilde{f}^\dagger_{\mathbf{r}\sigma} \tilde{f}_{\mathbf{r}'\sigma} + J_H \sum_{\langle \mathbf{r}\mathbf{r}'\rangle} \mathbf{S}_{\mathbf{r}} \cdot \mathbf{S}_{\mathbf{r}'} \Big] P_{\rm G}, \tag{2}$$

where t_f represents the nearest neighbor $\tilde{f}-$hole hopping amplitude and P_G denotes the Gutzwiller projection operator that forbids double occupancy at any given site. Finally, $H_{\rm hyb}$ is the term ($\sim V$) responsible for hybridization between the d and $f-$electrons. We begin by setting $V = 0$, which results in an enhanced $U_d(1) \times U_f(1)$ symmetry associated with the two conserved fermion numbers. Furthermore, as mentioned earlier, let us restrict ourselves to the regime where $U_{\rm ff} \gg U_{\rm df} \gg t_d \gg V$. In this limit and within the slave-boson treatment introduced earlier, the fermionic composite exciton hopping is given by,

$$t_{\rm CE} \sim \frac{t_d\, t_b}{U_{\rm df}}. \tag{3}$$

The holon hopping, t_b, can now be estimated using a self-consistent mean-field treatment of the Hamiltonian defined in Eq.2 above,

$$\begin{aligned} H_{{\rm t}_f\text{-}{\rm J}_H} \to & -t_f \sum_{\langle \mathbf{r}\mathbf{r}'\rangle} \langle \chi^\dagger_{\mathbf{r}\alpha}\, \chi_{\mathbf{r}'\alpha}\rangle b^\dagger_{\mathbf{r}} b_{\mathbf{r}'} \\ & - \sum_{\langle \mathbf{r}\mathbf{r}'\rangle} \Big(t_f \langle b^\dagger_{\mathbf{r}} b_{\mathbf{r}'}\rangle + J_H \langle \chi^\dagger_{\mathbf{r}\alpha}\, \chi_{\mathbf{r}'\alpha}\rangle \Big) \chi^\dagger_{\mathbf{r}\sigma} \chi_{\mathbf{r}'\sigma}, \end{aligned} \tag{4}$$

where we have ignored pairing terms for the spinon fields. The effective holon hopping will then simply be given by $t_b \sim t_f$ with an $O(1)$ coefficient, as long as the spinons are in a state (e.g. with a Fermi-surface) where $\langle \chi^\dagger_{\mathbf{k}} \chi_{\mathbf{k}}\rangle = n^\chi_{\mathbf{k}}$ is not flat as a function of $\mathbf{k}$. This leads to the estimate of the fCE hopping in Eq. 17. In the presence of a small but finite V, the resulting fCE and spinon bands hybridize and yield a semi "metal", as discussed in the main text. From the above equation, it is also possible to read off the self-consistently generated hopping for the spinons, t_χ.

SUPPLEMENTARY NOTE 2

Low-energy field theory: In the CEFL phase, the low-energy degrees of freedom are the fCE, the spinons and the gapped holons that are all minimally coupled to a dynamical gauge-field. The fCE and the spinons hybridize to yield a compensated semi-metal with 'particle-like' and 'hole-like' pockets. Let us then write down the resulting low-energy effective field theory. We continue to denote the resulting neutral fermions (which are superpositions of the fCE and the spinon) as $\psi_{\mathbf{k}\alpha,i}$, where we have introduced an additional label $i = 1, 2$; $i = 1$ for the particle-like pocket and $i = 2$ for the hole-like pocket. As described earlier, the ψ fermions couple minimally to a_μ and the non-relativistic b holons couple minimally to $\Delta a_\mu = a_\mu - A_\mu$. The Lagrangian

is given by

$$\mathcal{L} = \mathcal{L}_\psi + \mathcal{L}_b + \mathcal{L}_{\psi b} + \mathcal{L}_a, \quad (5)$$

$$\mathcal{L}_\psi = \psi^\dagger_{\alpha,i}(\partial_\tau - ia_0 - \mu_i)\psi_{\alpha,i} - \frac{1}{2m_i}\psi^\dagger_{\alpha,i}(-i\nabla - \mathbf{a})^2\psi_{\alpha,i}, \quad (6)$$

$$\mathcal{L}_b = b^*(\partial_\tau - i\Delta a_0 - \mu_b)b - \frac{1}{2m_b}b^*(-i\nabla - \Delta\mathbf{a})^2 b + \frac{u}{2}|b|^4 + ... \quad (7)$$

$$\mathcal{L}_{\psi b} = g_0\, |b|^2\, \psi^\dagger_{\alpha,i}\psi_{\alpha,i} + ..., \quad (8)$$

$$\mathcal{L}_a = \frac{1}{e^2}(\epsilon_{\mu\nu\lambda}\partial_\nu a_\lambda)^2. \quad (9)$$

The form of the above Lagrangian is similar to theories considered earlier in a different context [1]. Note that we have allowed density-density interaction terms between the holon density and composite exciton density in $\mathcal{L}_{\psi b}$ above; these terms are formally irrelevant close to the transition $\mu_b = 0$. $\mathcal{L}_a$ contains a Maxwell term for the emergent gauge-field, but as discussed in the methods section, integrating out the fermions leads to a landau-damped form of the propagator for the transverse-component of the gauge-field, D_{ij}, as in Eq.18. The time component of the gauge-field couples to the density and does not lead to any singular non-Fermi liquid behavior.

In addition to the properties of the holon self-energy, Σ_b, at $T = 0$ that we already described in the methods section, it is important to recall that at a finite temperature $T > 0$, $\Sigma_b(0,\vec{0}) \sim uT^{3/2}$ and therefore the properties associated with the holon are determined by an interplay of μ_b and the above "thermal" mass. In three-dimensions, there is no phase-transition associated with condensation of $\langle b\rangle$ at $T > 0$.

SUPPLEMENTARY NOTE 3

Ioffe-Larkin composition rules: We derive the Ioffe-Larkin sum rules [2] in this supplementary note. From the field theory description introduced in the previous supplementary note, integrating out the vector potential $\mathbf{a}$ leads to the constraint $\mathbf{j}_\psi + \mathbf{j_b} = \mathbf{0}$, where the $\mathbf{j}$ represent the respective currents, i.e. the holons and fermions can only move subject to this constraint. As a result of this constraint, the net electrical response of the system will correspond to the sequential (i.e. 'series') circuit of the two individual 'resistances'. Suppose we now integrate out the matter fields altogether and obtain an effective action purely in terms of the external and internal gauge fields,

$$S_{\rm eff}[\mathbf{A},\mathbf{a}] = \frac{1}{2}\int d^3\mathbf{r}\, d\tau \Big[\mathbf{a}\,\Pi_\psi\,\mathbf{a} + (\mathbf{A}-\mathbf{a})\,\Pi_b\,(\mathbf{A}-\mathbf{a}) + \mathbf{A}\,\Pi_0\,\mathbf{A} + ...\Big], \quad (10)$$

where we have restricted ourselves to the simplest quadratic action and ... denote additional contributions which may arise e.g. from the singular rearrangement of the Fermi surfaces in the presence of a finite $\mathbf{b} = \nabla\times\mathbf{a}$. The coefficients Π_ψ and Π_b denote the full response functions due to the neutral fermions and gapped holons respectively. We have also included Π_0, that arises from the trivial background for the sake of completeness. The response functions to leading order in small $\omega, \mathbf{q}$ are given by,

$$\Pi_\psi(\omega,\mathbf{q}) = i\omega\,\sigma_\psi(\omega,\mathbf{q}) - \chi_\psi\mathbf{q}^2, \quad (11)$$

$$\Pi_b(\omega,\mathbf{q}) = i\omega\,\sigma_b(\omega,\mathbf{q}) - \chi_b\mathbf{q}^2, \quad (12)$$

where $\sigma_{\psi,b}$ and $\chi_{\psi,b}$ denote the conductivities and diamagnetic susceptibilities respectively (Note that $\chi_{\psi,b} \equiv \mu^{-1}_{{\rm ce},b}$, as defined in the main text). Since $\mathbf{a}$ is a dynamical field and the path-integral sums over all allowed configurations of $\mathbf{a}$, we can integrate it out and determine the effective action purely in terms of the external vector potential, $\mathbf{A}$. The resulting action is given by,

$$S_{\rm eff}[\mathbf{A}] = \frac{1}{2}\int d^3\mathbf{r}\, d\tau\, \mathbf{A}\Big[\Pi_0 + \frac{\Pi_\psi\Pi_b}{\Pi_\psi + \Pi_b}\Big]\mathbf{A} \quad (13)$$

Let us now first focus on the limit of $\mathbf{q}\to 0$ and finite ω (i.e. there is a uniform electric field). In this limit, the net conductivity of the system can be read off from the second term above as,

$$\sigma(\omega) = \frac{\sigma_\psi(\omega)\,\sigma_b(\omega)}{\sigma_\psi(\omega) + \sigma_b(\omega)}, \quad (14)$$

which is the promised sequential response of the two resistors. Note that one can arrive at the same formula in terms of the shift of the gauge field $\mathbf{a}$ and its resulting 'backflow' effect. In the limit of $\omega \to 0$

(i.e. dc-limit) and at $T = 0$, $\text{Re}[\sigma_b] = 0$ and hence one obtains insulating response. The above expression is also the starting point for the discussion of optical conductivity [3], as explained in the main text.

Let us now consider the other case where $\omega \to 0$ and $\mathbf{q}$ is finite (i.e. there is a uniform magnetic-field). Here, one can read off the net diamagnetic response as,

$$\chi = \frac{\chi_\psi \chi_b}{\chi_\psi + \chi_b}, \tag{15}$$

in an electrical insulator. This is precisely the form of the diamagnetic response that one would obtain starting from the free-energy in Eq. 11 for $\mathbf{b} = \alpha \mathbf{B}$ (Eq.12); the resulting locking mechanism is then responsible for Landau quantization of the neutral Fermi-surface and quantum oscillations [4].

SUPPLEMENTARY NOTE 4

Comparison to other theoretical proposals for SmB_6: In this supplementary note, we compare our theoretical proposal for the CEFL with other proposals that have made an attempt to account for at least some of the anomalous features observed in experiments on SmB_6. The other proposals can be broadly classified as follows: (i) "Magnetic-breakdown" in a small-gap insulator [5, 6] - If the typical cyclotron energy, at large enough magnetic fields, is larger than the insulating gap, this can lead to Landau quantization and an analogue of breakdown effects (but as a function of energy). However within this picture, in the low temperature and zero magnetic field limit, the system is still an insulator and does not have any of the thermodynamic or optical signatures associated with the experimental observations described earlier. (ii) "Majorana" Fermi-surfaces [7, 8] - Within this picture, there is a Fermi-surface in the bulk, where the excitations are Majorana-like, instead of being complex fermions of the type we propose (i.e. fCE). In particular in the Majorana-based interpretation, the zero magnetic field state in the limit of zero temperatures is a superconductor, and not an electrical insulator. It is important to note that in an incompressible phase, all excitations carry a well defined charge; the Majorana fermions are objects where the anti-particle is identical to the particle itself and therefore carries no charge. Since there are no neutral fermionic excitations in a system described by an electronic Hilbert space in the UV, these objects can only emerge in the IR and be necessarily non-local. The only known route of doing this theoretically is to couple them to an emergent gauge-field; the non-local Majorana fermions can be coupled to a discrete (e.g. in the simplest case a Z_2) gauge field. In three dimensions, there is then necessarily a finite temperature phase transition associated with transition into the phase with deconfined, non-local Majorana excitations. This should be seen e.g. as a divergence in the specific heat at the transition. Within our framework, there is no finite temperature phase transition into the CEFL phase with a deconfined $U(1)$ gauge-field, even in three dimensions. (iii) Gapped (conventional) excitonic insulators [9] - As a result of a large joint density of states, it is possible that the system is close to a conventional (bosonic) excitonic instability with a finite momentum, $\mathbf{Q}$ (governed by details of the band-structure), but with a small gap, Δ. If the energy gap is small along a ring of momentum around $\mathbf{Q}$ (e.g. like a roton), in the limit of temperatures larger than the gap it can give rise to power-law features in some thermodynamic properties (as opposed to exponential in Δ/T). However, one concern is that this requires some fine-tuning as Δ needs to be small enough such that the experimentally accessible temperatures are already larger than the gap. At the same time the system needs to avoid undergoing the excitonic instability into a density-wave state (with wavevector $\mathbf{Q}$), since there is no experimental evidence of the system being close to any density-wave instability.

SUPPLEMENTARY REFERENCES

[1] T. Senthil, M. Vojta, and S. Sachdev, "Weak magnetism and non-fermi liquids near heavy-fermion critical points," Phys. Rev. B **69**, 035111 (2004).

[2] L. B. Ioffe and A. I. Larkin, "Gapless fermions and gauge fields in dielectrics," Phys. Rev. B **39**, 8988 (1989).

[3] T.-K. Ng and P. A. Lee, "Power-law conductivity inside the mott gap: Application to $\kappa-(\mathrm{BEDT-TTF})_2\mathrm{cu}_2(\mathrm{CN})_3$," Phys. Rev. Lett. **99**, 156402 (2007).

[4] I. Sodemann, D. Chowdhury, and T. Senthil, "Quantum oscillations in insulators with neutral fermi surfaces," Phys. Rev. B **97**, 045152 (2018).

[5] J. Knolle and N. R. Cooper, "Quantum oscillations without a fermi surface and the anomalous de haas˘van alphen effect," Phys. Rev. Lett. **115**, 146401 (2015).

[6] L. Zhang, X.-Y. Song, and F. Wang, "Quantum oscillation in narrow-gap topological insulators," Phys. Rev. Lett. **116**, 046404 (2016).

[7] G. Baskaran, "Majorana Fermi Sea in Insulating SmB6: A proposal and a Theory of Quantum Oscillations in Kondo Insulators," ArXiv e-prints (2015), arXiv:1507.03477 [cond-mat.str-el].

[8] O. Erten, P.-Y. Chang, P. Coleman, and A. M. Tsvelik, "Skyrme insulators: Insulators at the brink of superconductivity," Phys. Rev. Lett. **119**, 057603 (2017).

[9] J. Knolle and N. R. Cooper, "Excitons in topological kondo insulators: Theory of thermodynamic and transport anomalies in smb_6," Phys. Rev. Lett. **118**, 096604 (2017).
